# Application of pyrite and chalcopyrite as sensor electrode for amperometric detection and measurement of hydrogen peroxide

**DOI:** 10.1039/c7ra13628e

**Published:** 2018-01-30

**Authors:** Y. Wang, K. J. Zhao, D. P. Tao, F. G. Zhai, H. B. Yang, Z. Q. Zhang

**Affiliations:** School of Chemical Engineering, University of Science and Technology Liaoning 185 Qianshan Middle Road, High-tech zone Anshan Liaoning 114501 China zhangzhiqiang@ustl.edu.cn; School of Mining Engineering, University of Science and Technology Liaoning 185 Qianshan Middle Road, High-tech zone Anshan Liaoning 114501 China dptao@qq.com

## Abstract

The sensing performance of solid-state amperometric sensors based on natural sulfide minerals, *i.e.*, pyrite and chalcopyrite, has been characterized for the detection and measurement of hydrogen peroxide (H_2_O_2_) in aqueous medium. The sensors showed a wide linear relationship range between response current and the concentration of H_2_O_2_ from 1.0 × 10^−5^ mol L^−1^ to 1.0 × 10^−2^ mol L^−1^ and 1.0 × 10^−4^ mol L^−1^ to 3.0 × 10^−2^ mol L^−1^ for pyrite and chalcopyrite, respectively. The limit of detection (LOD) was as low as 8.6 × 10^−6^ mol L^−1^ and 5.2 × 10^−5^ mol L^−1^ (S/N = 3), respectively. The electrodes exhibited great sensitivity, repeatability and short response time (less than 5 s). The results show that pyrite and chalcopyrite can be used as a natural, low cost, reliable and sensitive sensor for hydrogen peroxide detection, creating a new and high value application for the sulfide minerals.

## Abstract 2

The sensing performance of solid-state amperometric sensors based on natural sulfide minerals, *i.e.*, pyrite and chalcopyrite, has been characterized for the detection and measurement of hydrogen peroxide (H_2_O_2_) in aqueous medium.

## Graphical abstract

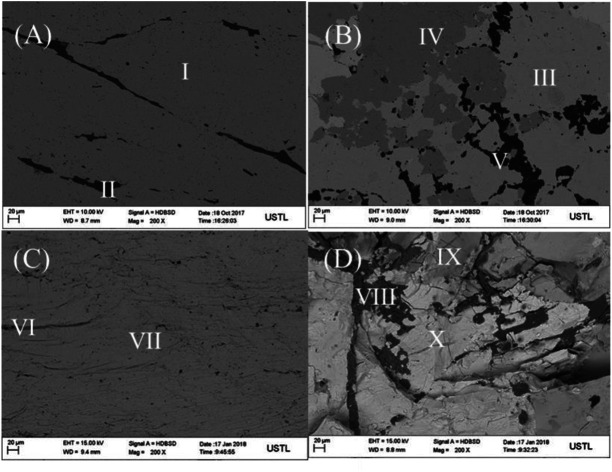

## Introduction

Hydrogen peroxide (H_2_O_2_) is widely present in nature, particularly in waterways and various life systems. It is not only a product of the reactions catalyzed by various oxidase enzymes, but also an essential compound in industrial, clinical, pharmaceutical and environmental analyses.^1,2^ The sensitive, accurate, and low-cost determination of H_2_O_2_ is essential for industrial processes and clinical research. There are a number of methods for H_2_O_2_ detection and measurement, including spectrophotometry,^3^ titrimetry,^4^ X-ray absorption,^5^ chemiluminescence^6^ and fluorometric methods.^7^ These methods usually require expensive equipment and reagents, complicated and time-consuming procedures and skillful operators. Therefore, the electrochemical technique for H_2_O_2_ detection becomes one of the most attractive alternatives due to its simplicity, high sensitivity and selectivity.^8–11^ It has proved to be an effective and simple technique for H_2_O_2_ determination.^12^

Conventional electrochemical technique for H_2_O_2_ detection and measurements is usually carried out using enzyme immobilization modified electrodes, which have gained great interest due to its unique advantage in sensitivity and selectivity.^13,14^ However, the enzyme-based sensors often lack of acceptable stability due to the inherent characteristics of enzymes the activity of which can readily be affected by temperature, pH, humidity, and toxic chemicals.^15,16^ Therefore, much attention has recently been paid to the development of non-enzymatic electrochemical H_2_O_2_ sensors for their simplicity, high reliability and sensitivity and low cost.^17–19^ Numerous functional materials were used for the non-enzymatic sensing of H_2_O_2_, including nanostructured materials,^20–22^ ionic liquid,^23^ polymer,^24^ sol–gel^25^ and ceramic matrix.^26,27^ In order to develop a better technique for detection and measurement of H_2_O_2_, electrodes made of natural pyrite and chalcopyrite have been investigated in this study for their feasibility to function as a sensor for H_2_O_2_ in aqueous medium.

Pyrite and chalcopyrite represent the Earth's most abundant and widespread sulfide minerals. Pyrite, with the formula of FeS_2_, is a Fe(ii) polysulfide with a cubic NaCl-type crystalline structure. It is the most thermodynamically stable iron sulfide found in the nature. Over the past decades, the electrochemistry of pyrite has been studied extensively.^28–31^ As the most abundant copper mineral, chalcopyrite (CuFeS_2_) is the most economically important copper resource and is always found in association with pyrite. The selective separation of chalcopyrite from pyrite is very difficult because of several electrochemical interactions that occur at the surface of minerals during grinding.^32^ New applications of pyrite and chalcopyrite are of great importance. Due to their excellent characteristics such as semiconductivity, non-toxicity, and availability in the nature pyrite and chalcopyrite have recently been used in various electrochemical applications in the form of solid state sensor materials.^33–36^

In this study, the natural minerals (pyrite and chalcopyrite) were used as the working electrode to detect the H_2_O_2_ in aqueous solution. The main purpose of this study was to further explore analytical applications of pyrite and chalcopyrite electrodes, with a focus on developing an inexpensive, rapid and convenient method for the determination of biologically important compound, H_2_O_2_. It has been found that under the optimum operational conditions, the pyrite and chalcopyrite based sensors showed good sensitivity, rapid response time and excellent operational stability for the detection and measurement of hydrogen peroxide. Compared with traditional non-enzyme biosensor that works in alkaline solution, the pyrite and chalcopyrite sensors can detect H_2_O_2_ not only in alkaline solution, but also in acidic and neutral environment with Britton–Robinson buffer, which is an amazing feature for a new analytical sensor developed from different material. This unique characteristic of mineral sensors will lead to more extensive applications of H_2_O_2_ sensors in various industries.

## Experimental

### Reagents and materials

The experiments were carried out with samples of natural pyrite and chalcopyrite (from Shanxi province, China) without further purification. The pyrite and chalcopyrite samples were first cut into about 5 mm × 5 mm × 5 mm in size in the university laboratory. The processed sulfide mineral samples were then sent to Tianjin AIDAhengsheng Science-Technology Development Co., Ltd. to fabricate the sensor electrodes. The fabrication process is described briefly as follows: the column-shaped mineral electrode sample was covered by Teflon with only two ends exposed to air. One end was connected to a copper wire and the other end was inserted into the solution to detect H_2_O_2_. The copper wire was connected to the electrochemical analyzer. The working area was 0.20 cm^2^ for both pyrite and chalcopyrite electrodes. Prior to each experiment, the working surface of an electrode was polished using alumina powder (0.05 μm) for 30 s to obtain a shiny new surface, rinsed with deionized water and dried at the room temperature.

Uric acid (UA), glucose, fructose, sodium hydroxide (NaOH), phosphoric acid, glacial acetic acid, boric acid and hydrogen peroxide (H_2_O_2_) were purchased from Sinopharm Chemical Reagent Co., Ltd. A 0.1 M Britton–Robinson buffer (BR, prepared by mixing phosphoric acid, glacial acetic acid and boric acid) was used to prepare electrolyte for the acidic solution. The neutral and alkaline solutions were adjusted by blending the mixed acid and NaOH. All chemicals were of analytical grade and were used without further purification.

### Apparatus

The morphology of the minerals was observed using the field-emission scanning electron microscope (FE-SEM) (ΣIGMA-HD, ZEISS). The chemical components of pyrite and chalcopyrite were obtained using an X-ray fluorescence analyzer EDX 8300 manufactured by Suzhou Precision Instrument Co., Ltd. Electrochemical measurements were performed with a CHI 750D workstation (Shanghai Chenhua, China). A conventional three electrode system with pyrite or chalcopyrite as working electrode, a thin Pt wire as counter electrode and Ag/AgCl (sat. KCl) as reference electrode was employed in this study. All measurements were performed in air at room temperature of approximately 20 °C.

## Results and discussion

### Characterization of pyrite and chalcopyrite

**Fig. 1:**
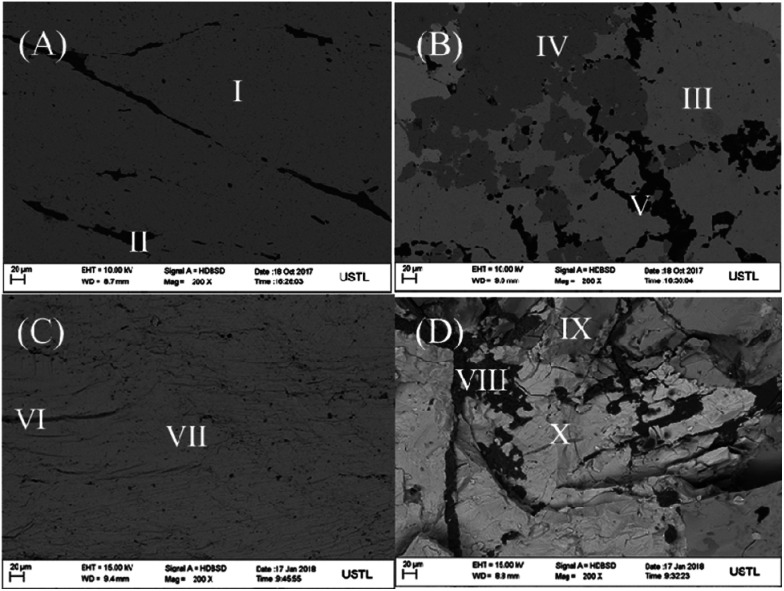
The SEM images of pyrite electrode surface (A), chalcopyrite electrode surface (B), pyrite middle section (C) and chalcopyrite middle section (D).

Field emission scanning electron micrograph (FE-SEM) was used to investigate the structure and morphology of pyrite electrode surface (A), chalcopyrite electrode surface (B), pyrite middle section (C) and chalcopyrite middle section (D) as shown in Fig. 1. The black parts (II, V, VI, VIII) in Fig. 1 are attributed to the impurities. Since chalcopyrite always coexists with pyrite in natural minerals, it is not unusual to observe the dark grey parts (I, IV, VII, IX) which represent the pyrite mineral. The light grey (part III, X) is the morphology of chalcopyrite. The SEM characterization was performed with the middle section of mineral sample after fracture and the results is in good agreement with pyrite and chalcopyrite electrode surfaces, implying the uniformity in composition of the minerals along the electrode length. The FE-SEM energy spectra showed that the FeS_2_ content of pyrite and chalcopyrite are 91.2% and 63.6%, respectively. Comparing the SEM image and energy spectrum indicates that half of chalcopyrite mineral is composed of pyrite.

**Fig. 2:**
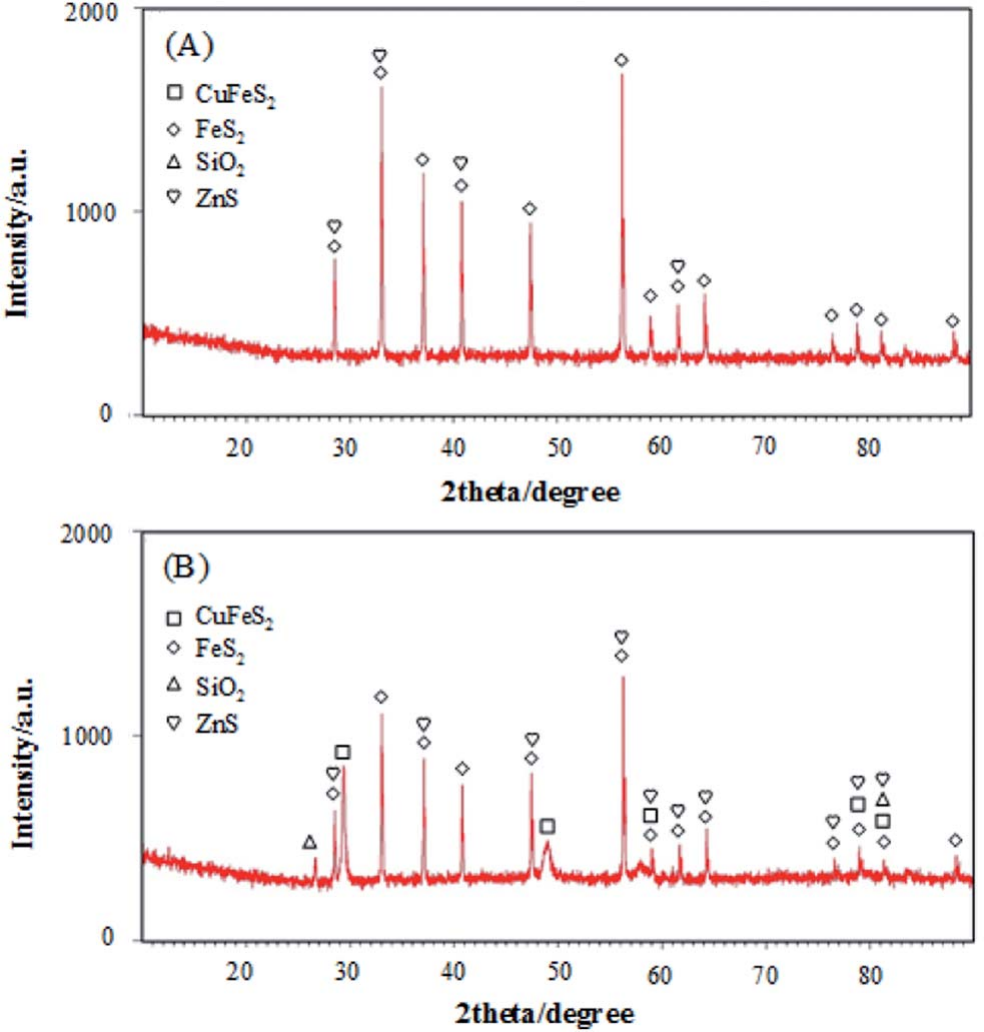
The XRD patterns of pyrite (a) and chalcopyrite (b).

The phase characteristics and structures of pyrite and chalcopyrite were identified from the X-ray diffraction (XRD) patterns shown in Fig. 2. The broad diffraction peaks observed in Fig. 2A can be assigned to FeS_2_ structure which is the main content of pyrite sample. FeS_2_ is also a major component in Fig. 2B, confirming pyrite is closely associated with chalcopyrite. The five diffraction peaks observed with 2*θ* values of 29.44, 49.11, 59.06, 79.06 and 81.52 are attributed to CuFeS_2_. Fig. 2 suggests both pyrite and chalcopyrite contain impurities such as SiO_2_ and ZnS which is in accordance with the XRF data shown below.

To quantify the components of pyrite and chalcopyrite minerals, X-ray fluorescence (XRF) analysis was carried out and the results are shown in Table 1. The impurities in pyrite can introduce significant variations in its bulk semi-conducting properties which can directly affect the reactivity of pyrite surfaces.^37^Table 1 shows that the powder pyrite sample contained a very low content of Cu element. Thus, iron sulfide FeS_2_ plays a dominant role in the detection of H_2_O_2_. The XRF data obtained with pyrite powder showed a lower purity than that of the FE-SEM energy spectra. It is believed that the purity of electrode surface used to detect H_2_O_2_ was enhanced to some extent by polishing and soaking in alkaline solutions.

**Table 1:** XRF analysis of pyrite and chalcopyrite powder

Mineral	Fe	Cu	S	Impurities
Pyrite	33.05	0.11	10.95	10.47
Chalcopyrite	22.16	11.72	8.20	10.45

### Electrocatalytic performance of H_2_O_2_ on pyrite and chalcopyrite

**Fig. 3:**
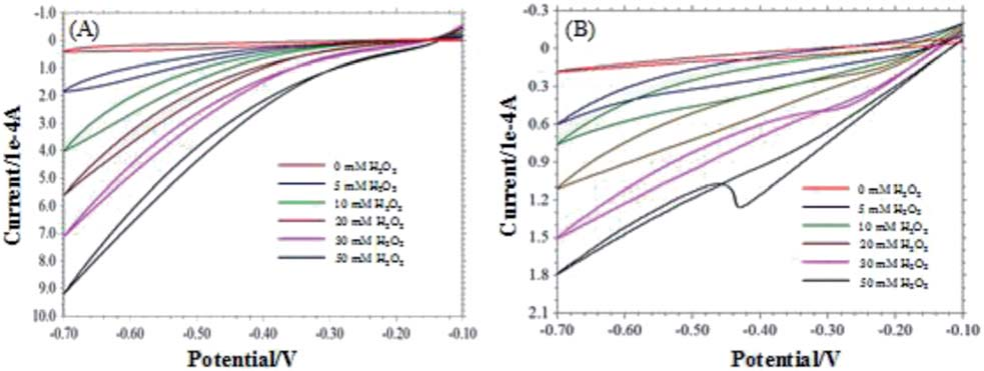
CVs of pyrite (A) and chalcopyrite (B) in the different concentrations of H_2_O_2_ (0–50 mM).

The CVs of the pyrite and chalcopyrite with and without H_2_O_2_ in 0.1 M NaOH are shown in Fig. 3A and B. The reduction peak current increased with increasing the concentration of H_2_O_2_, indicating the pyrite and chalcopyrite based sensors have good sensitivity for H_2_O_2_. However, the sensitivity of chalcopyrite is lower than that of pyrite. The large content of impurity in chalcopyrite resulted in the irregular CV curves and lower sensitivity.

**Fig. 4:**
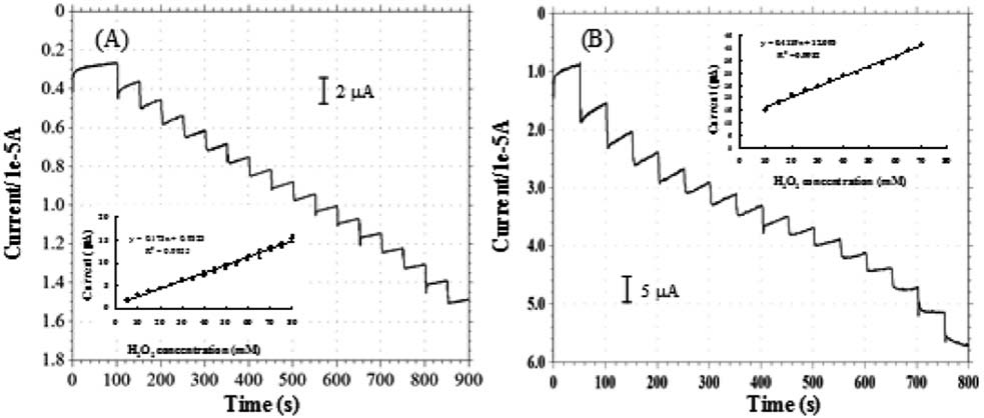
Amperometric responses of H_2_O_2_ on pyrite ((A), applied potential −0.2 V, [H_2_O_2_] = 5 mM) and chalcopyrite ((B), applied potential −0.4 V, [H_2_O_2_] = 5 mM).

Fig. 4A shows the typical current plot for the pyrite based sensor on successive additions of 5 mM H_2_O_2_ in 25 mM NaOH under stirring at the working potential of −0.2 V. Fig. 4B is the typical current response for chalcopyrite electrode on successive additions of 5 mM H_2_O_2_ in 25 mM NaOH at the working potential of −0.4 V. Well-defined current responses for H_2_O_2_ were obtained at both pyrite and chalcopyrite electrodes. The pyrite sensor exhibited good repeatability with a relative standard deviation (RSD) of 4.2% for 10 successive measurements. The chalcopyrite sensor also showed rather good repeatability with a RSD of 5.3% for 8 incremental additions of H_2_O_2_. These results are better than certain H_2_O_2_ biosensors that undergo the complicated fabrication procedures.^39,40^ The response time is one of the most important parameters for describing sensor characteristics.^41^ The response current achieved the steady state in less than 3 seconds for pyrite and within 4 seconds for chalcopyrite, representing rapid response rates for natural minerals as compared with other materials such as ceramic carbon composite,^42^ carbon felt,^11^ glassy carbon,^43^ iron nanoparticles,^44^ and carbon nanotubes.^45^ Based on the above results it is reasonable to conclude that pyrite and chalcopyrite are excellent natural mineral sensors for detection and measurements of H_2_O_2_.

**Fig. 5:**
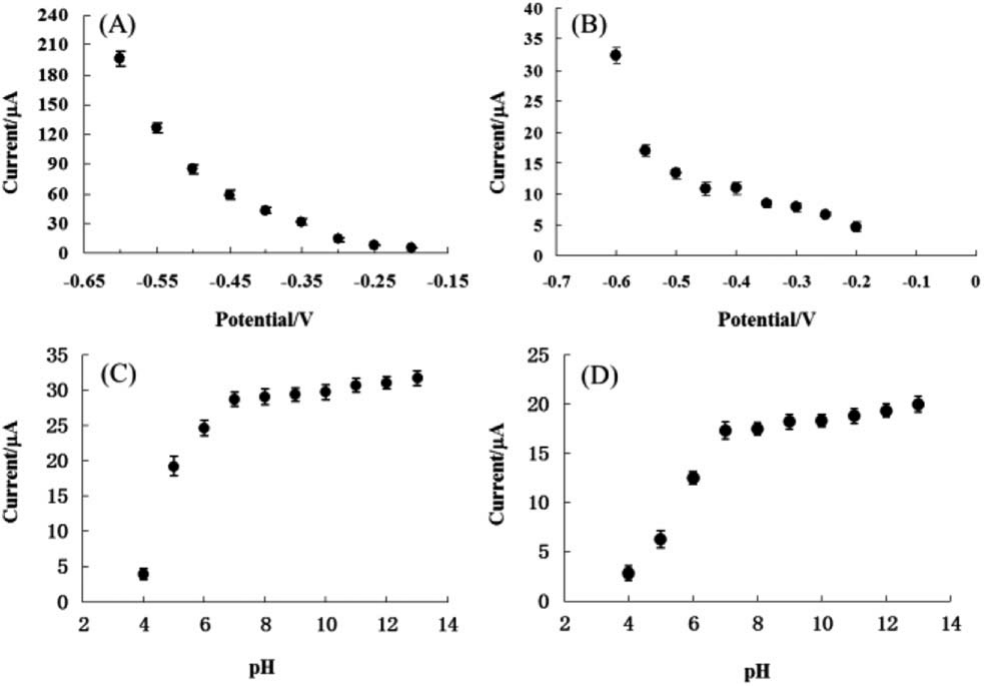
(A) Effect of applied potential on the amperometric response of pyrite in the presence of 10 mM H_2_O_2_ in 0.1 M NaOH. (B) Effect of applied potential on the amperometric response of chalcopyrite in the presence of 10 mM H_2_O_2_ in 0.1 M NaOH. (C) Effect of pH on the amperometric response of pyrite in the presence of 10 mM H_2_O_2_, applied potential −0.4 V. (D) Effect of pH on the amperometric response of chalcopyrite in the presence of 10 mM H_2_O_2_, applied potential −0.4 V.

The same component FeS_2_ exists in both pyrite and chalcopyrite minerals. It is speculated that the electro-catalytic reaction happened between FeS_2_ and H_2_O_2_ in the two electrodes. The results in Fig. 5C and D indicate that even in acidic and neutral solution, pyrite and chalcopyrite still can detect H_2_O_2_ in the negative potential range. This suggests hydroxyl group (OH–) is not essential for the electro-catalytic reaction. FeS_2_ was oxidized by H_2_O_2_ to produce Fe^3+^ and S^0^. Previous studies have found that Fe^3+^ can be reduced to Fe^2+^ at −0.4 V ^30^ and the reduction of S^0^ to HS^−^ happened at −0.6 V.^38^Fig. 3 reveals that the reduction current increased with decreasing the applied potential, as more elemental sulphur was reduced to HS^−^ at lower potentials.

In order to identify the optimum conditions for pyrite and chalcopyrite electrodes used for detection of H_2_O_2_, the applied potential and the pH dependency of electrolyte were varied in aqueous solutions with 10 mM H_2_O_2_. The response currents were measured as a function of applied potentials between −0.2 V and −0.6 V with a step of 0.05 V and the results are shown in Fig. 5A and B for pyrite and chalcopyrite sensors, respectively. The reduction current of H_2_O_2_ increased consistently with decreasing the applied potential. Because the baseline also increased significantly with decreasing the applied potential, the lower potential value was employed in the subsequent experiments to avoid the reduction of soluble oxygen in solution. The effect on reduction current of pH from 4 to 13 was also studied and the results are shown in Fig. 5C and D for pyrite and chalcopyrite, respectively. The amperometric response increased from pH 4 to pH 7, reached a plateau at pH 7, and maintained essentially constant until pH 13 for both pyrite and chalcopyrite. This wide pH range is preferred not only for the commercial applications, but also for future study in combination with enzymes. These results show that the OH^−^ group is not essential for the detection of H_2_O_2_. It should be pointed out that strongly alkaline solution is not desirable either from the viewpoint of sensor's practical applications. The finding is very encouraging for commercial applications of mineral sensors in complicated environment although the operational stability is not satisfying in the neutral and acid conditions with the current design of electrodes and refinement in electrode fabrication is needed for better sensing performance.

**Fig. 6:**
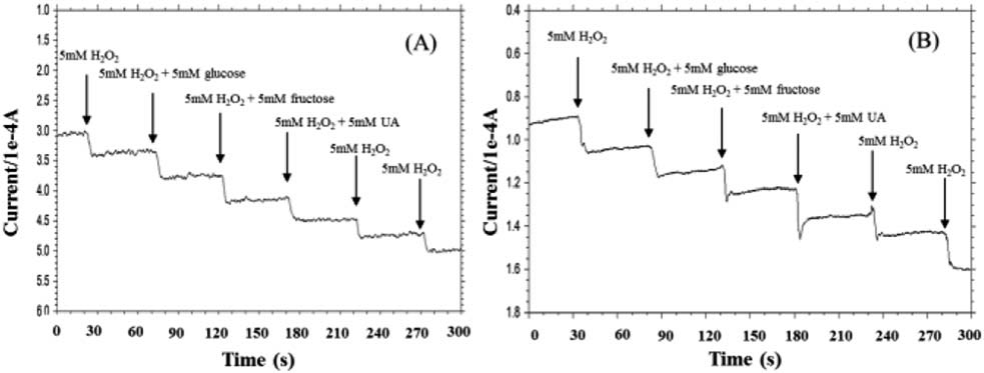
Amperometric responses of the pyrite (A) and chalcopyrite (B) electrodes for the successive additions of 5 mM H_2_O_2_, the mixture of 5 mM H_2_O_2_ and glucose, the mixture of 5 mM H_2_O_2_ and fructose, the mixture of 5 mM H_2_O_2_ and UA, 5 mM H_2_O_2_ (current–time response measured in 0.1 M NaOH at −0.6 V).

Selectivity is another important parameter for non-enzymatic H_2_O_2_ sensor since a good selectivity ensures high accuracy.^46^ To investigate the selectivity of pyrite and chalcopyrite for H_2_O_2_, the effect of interfering reagents on the response current has been studied. The typical amperometric response on successive additions of 5 mM H_2_O_2_, and 5 mM interference species (UA, glucose, fructose) under stirring is shown in Fig. 6. It is important to minimize the effect of interfering species possibly existing in real aqueous solutions for practical applications of amperometric sensors. As shown in Fig. 6, a clear current step was observed with the addition of H_2_O_2_ and negligible effects on the current response were observed when glucose, fructose and UA were added into the solution, demonstrating that the natural minerals can be used to detect H_2_O_2_ in the presence of these interferents at the same concentration. The results seemed acceptable in comparison with conventional H_2_O_2_-detected biosensors.^47–49^ The good anti-interference ability may largely be attributed to the low working potential used in the determination of H_2_O_2_.^50^

**Fig. 7:**
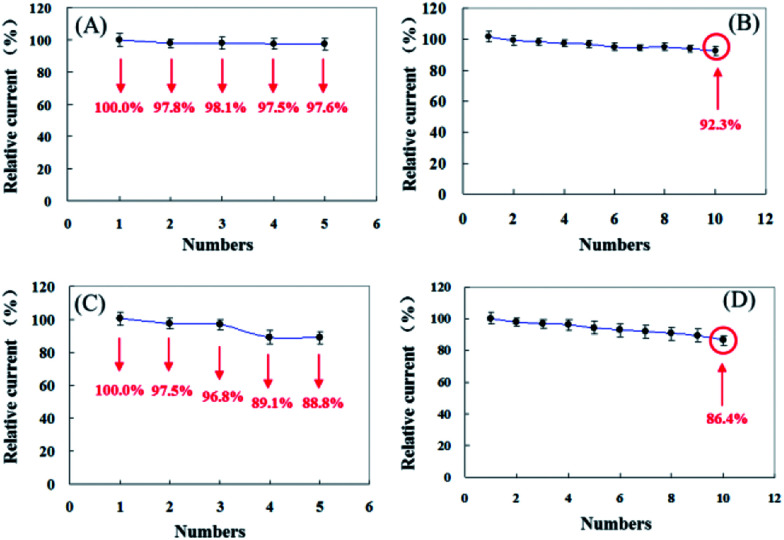
The reproducibility and repeatability of pyrite (A, B) and chalcopyrite (C, D).

The repeatability and reproducibility of pyrite and chalcopyrite sensors were examined by detecting 5 mM H_2_O_2_ five times with an interval of 24 hours and the results are shown in Fig. 7. In comparison pyrite shows better repeatability and reproducibility than chalcopyrite sensor. The relative standard deviation (RSD) of repeatability and reproducibility is 2.77% and 0.95% for pyrite and 4.27% and 4.98% for chalcopyrite, which represent very good performance, especially for natural mineral sensors.

**Fig. 8:**
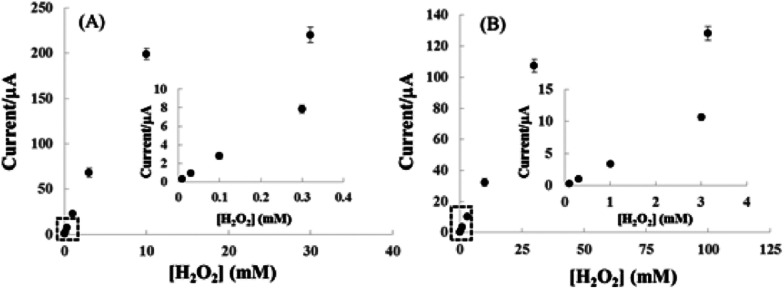
Calibration plots of H_2_O_2_ obtained with pyrite (A) and chalcopyrite (B) sensors. The insets are enlargement of lower concentration range of the calibration plots of pyrite and chalcopyrite. Applied potential is −0.6 V *vs.* Ag/AgCl in 25 mM NaOH aqueous solution.

Fig. 8 shows the correlation between cathodic peak current and H_2_O_2_ concentration for pyrite (A) and chalcopyrite (B) electrode in 25 mM NaOH aqueous solution at the applied potential of −0.6 V. The pyrite and chalcopyrite sensors show a wide range of linear relationship between the response current and H_2_O_2_ concentration from 1.0 × 10^−5^ mol L^−1^ to 1.0 × 10^−2^ mol L^−1^ with a correlation coefficient of 0.997 and from 1.0 × 10^−4^ mol L^−1^ to 3.0 × 10^−2^ mol L^−1^ with a correlation coefficient of 0.991 for pyrite and chalcopyrite, respectively. The limit of detection (LOD) was as low as 8.6 × 10^−6^ mol L^−1^, with a sensitivity of 19.61 μA mM^−1^ for pyrite (S/N = 3) whereas the LOD was 5.2 × 10^−5^ mol L^−1^ with a sensitivity of 3.21 μA mM^−1^ for chalcopyrite (S/N = 3). In comparison with other H_2_O_2_-based sensors including enzyme and non-enzyme system shown in Table 2, the characteristics of pyrite and chalcopyrite sensors were rather impressive, especially considering the natural minerals were directly used without any purification. Although certain nanomaterial modified electrodes possess better performance but they are much more costly and require a complex fabrication process.

**Table 2:** Comparison between the sensors investigated in this study and other H_2_O_2_ sensors^a^

Electrode material	Linear range (mol L^−1^)	LOD (μmol L^−1^)	Reference
HRP/SGCCN/GCE	5.0 × 10^−4^ to 1.0 × 10^−2^	12.89	51
HRP/sol–gel/MWCNT/GCE	7.0 × 10^−5^ to 3.0 × 10^−3^	14	52
GO–Ag nanocomposite	1.0 × 10^−4^ to 1.1 × 10^−3^	28.3	53
nanoCoPc-Gr	1.0 × 10^−5^ to 6.0 × 10^−4^	10.1	54
Cu_2_O/Au	2.5 × 10^−7^ to 5.0 × 10^−3^	0.12	55
CuNi/MWCNT/GCE	1.0 × 10^−7^ to 5.0 × 10^−3^	0.0025	56
υ-CuNWs	5.0 × 10^−7^ to 8.0 × 10^−4^	0.4	57
pTB–HRP–GOx/RGO	−3.0 × 10^−5^	0.2	58
Pyrite	1.0 × 10^−5^ to 1.0 × 10^−2^	8.6	This work
Chalcopyrite	1.0 × 10^−4^ to 3.0 × 10^−2^	52	This work

a SGCCN sol–gel-derived ceramic-carbon nanotube, GCE glass carbon electrode, MWCNT multiwalled carbon nanotubes, GO graphene oxide, nanoCoPc cobalt phthalocyanine nanorods Gr graphene, Cu_2_O cuprous oxide nanowires, CuNi copper/nickel, υ-CuNWs vertically aligned copper nanowires, pTB–HRP–GOx/RGO poly(toluidine blue)–horseradish peroxidase–glucose oxidase/reduced graphene oxide.

To further investigate the selectivity and applicability of pyrite and chalcopyrite towards H_2_O_2_, disinfector samples and drinking water were used as real samples. The results are presented in Table 3. The results were consistent with the conventional potassium permanganate titration method.^59^ RSD and recovery were less than 3.5% and 98.0–103.8%, respectively for pyrite sensor and less than 4.8% and 97.4–107.0% for chalcopyrite sensor, suggesting that both pyrite and chalcopyrite can be used to detect and measure the concentration of H_2_O_2_ in real samples with reasonably good performance.

**Table 3:** Pyrite and chalcopyrite sensors applied to drinking water and H_2_O_2_ in disinfector samples

Sensors	Samples	Number	Measured by proposed H_2_O_2_ biosensor					Measured by KMnO_4_-titrated method
			Detected (μM)	Added (μM)	Found (μM)	RSD^a^ (%)	Recovery (%)	
Pyrite	Drinking water	1	Not found	50	51.9	3.5	103.8	Not found
		2	Not found	100	98.0	2.6	98.0	Not found
		3	Not found	200	202.1	2.5	101.1	Not found
	Disinfector samples	1	100.2	50	151.9	2.1	101.1	99.9
		2	200.5	100	302.3	2.7	100.6	200.2
		3	299.3	200	494.5	1.9	99.0	299.5
Chalcopyrite	Drinking water	1	Not found	50	53.5	4.8	107.0	Not found
		2	Not found	100	97.4	3.7	97.4	Not found
		3	Not found	200	203.9	4.0	102.0	Not found
	Disinfector samples	1	99.6	50	153.1	3.8	102.3	99.3
		2	200.1	100	305.0	3.5	101.6	199.8
		3	300.5	200	492.0	4.2	98.3	300.1

a RSD (%) calculated from three separate experiments.

## Conclusions

Electrochemical characteristics of the solid-state amperometric sensors based on the natural sulfide minerals of pyrite and chalcopyrite are investigated in aqueous medium. The electrodes were conveniently prepared using inexpensive natural sulfide minerals that originated from the natural environment. In 25 mM NaOH solution at −0.6 V (*vs.* Ag/AgCl) the pyrite electrode and chalcopyrite electrode displayed a current response that is in linear relationship with H_2_O_2_ concentration in a wide range from 1.0 × 10^−5^ mol L^−1^ to 1.0 × 10^−2^ mol L^−1^ and 1.0 × 10^−4^ mol L^−1^ to 3.0 × 10^−2^ mol L^−1^, respectively. Moreover, the sulfide mineral electrodes exhibited good repeatability and rapid response time (about 3–4 s). The pyrite and chalcopyrite can be used over a wide pH range. Based on this study, it is believed that other sulfide minerals may also work as the sensor for the detection of H_2_O_2_. Future studies will be focused on practical usage of natural sulfide minerals as sensitive electrochemical amperometric sensors in aqueous and non-aqueous solution for detection and concentration measurements of some chemical compounds important for pharmaceutical and environmental applications.

## Conflicts of interest

There are no conflicts to declare.

## Supplementary Material
